# Diagnosis and treatment of dehydration after stroke: A synthesis of existing evidence

**DOI:** 10.12968/bjnn.2023.19.Sup5.S24

**Published:** 2023-10-01

**Authors:** Colette Miller, Alison S.R. Mcloughlin, Valerio Benedetto, Danielle L. Christian, Stephanie P. Jones, Eleanor Smith, Caroline L. Watkins

**Affiliations:** 1 IMPlementation and Capacity building Team (IMPaCT), Applied Health Research Hub, University of Central of Central Lancashire (UCLan), Preston; 2 Stroke Research Team, School of Nursing and Midwifery, UCLan, Preston; 3 NIHR Applied Research Collaboration North West Coast (ARC NWC), UCLan, Preston; 4 Methodological Innovation, Development, Adaptation & Support Theme (MIDAS), Applied Health Research Hub, University of Central

**Keywords:** Stroke, Dehydration, Diagnosis, Assessment, Management, Systematic Review

## Abstract

Dehydration after stroke is associated with poor health outcomes, increased mortality, and poses a significant economic burden to health services. Yet research suggests that monitoring and assessment of hydration status is not routinely undertaken. In this commentary, we critically appraise a systematic review which aimed to synthesise the existing evidence regarding diagnosis and treatment of dehydration after stroke. The review discusses common measures of dehydration, describes studies evaluating rehydration treatments, and highlights the link between dehydration and poorer health outcomes in both human and animal studies. The reviewers suggest, future research should focus on determining a single, validated, objective measure to clinically diagnose dehydration in stroke patients. Research designs should include clearly defined patient characteristics, type and severity of stroke, and type and time point of dehydration measurement, to enable comparison between studies. Management of hydration status is a crucial element of acute stroke care which should be routinely practiced.

## Introduction

Globally, there are around 80 million individuals who have experienced a stroke, and it is estimated that over 13 million new cases of stroke occur each year (Johnson et al. 2019). It is estimated that around 36% of stroke patients are dehydrated upon admission, and 62% will become dehydrated during their hospital stay (Rowat et al. 2012). Dehydration after stroke is associated with increased mortality, poor health outcomes, and poses a significant economic burden to health services (Edmonds et al. 2021; Bhalla et al. 2000; Kelly et al. 2004). Ensuring sufficient hydration during (and following) the acute phase of a stroke offers benefits in that it can mitigate complications including infections, constipation, delirium, and venous thromboembolism (Kelly et al. 2004; Miller et al. 2023; Stotts and Hopf 2003; Visvanathan et al. 2015).

Despite the importance of adequate hydration after stroke being emphasised in international clinical practice guidelines (Intercollegiate Stroke Working Party 2023; Powers et al. 2018; Stroke Foundation 2023), research suggests that monitoring and assessment of hydration status is not routinely completed, and consequently dehydration is often recognised as a result of tests for other clinical conditions and complications (Watkins et al. 2017; Mullins, 2021). The reasons for this disparity between guidelines and practice are not completely understood but may be explained in part by a lack of consensus regarding definitions, diagnosis, and treatment of dehydration (Lacey et al. 2019). In the most recent systematic review on this specific topic, Bahouth and colleagues aimed to identify and synthesise the existing evidence regarding diagnosis and treatment of dehydration after stroke to inform future research and practice (Bahouth et al. 2018).

### Aim of commentary

This commentary aims to critically appraise the methods used within the review by Bahouth et al. (2018) and to expand on the review findings in the context of clinical practice.

## Methods of Bahouth et al (2018)

Multiple databases were used in the review including PubMed, CINAHL, Cochrane and Scopus. Search terms included “hydration”, “dehydration”, “blood viscosity”, "volume contraction”, “hypertonicity”, “thirst” and “haemodilution”. Studies were included if they were published between the years 1997 and 2017. The authors chose to commence the search from 1997 as this marked a significant transformation in the treatment of acute stroke (thrombolytic therapy for acute stroke patients) (Bahouth et al. 2018). In addition to these, the reviewers conducted backward citation searches, as well as including a pre-1997 seminal study which investigated dehydration and stroke.

Only studies which examined hydration status in hospitalised patients with first time ischaemic stroke were included. The study team only reviewed papers written in English and excluded both research around dehydration linked to difficulties with swallowing, and studies focusing on dehydration occurring beyond the acute phase of stroke, defined by the authors as “the immediate post-stroke period”.

The authors did not indicate how many reviewers were involved in the title/abstract and full-text screening or in the data extraction. One reviewer used the Quality Assessment Tool for Quantitative Studies (Bahouth et al. 2018; Ciliska et al. 1998) to investigate the potential bias of the included studies. No indication was provided on the method of synthesis.

## Results

There was variation between the review aims and the reported results, but this may be due to the difficulties in combining the varied literature around this topic. There were several inconsistencies in the reporting of the total number of studies included across the review and more details are provided in the relevant results sections. Quality assessment was only reported for the 23 studies included in the data tables, of which 7 (30%) were reported as moderate, with the remaining 16 (70%) weak indicating an overall low quality of evidence (Bahouth et al. 2018).

### Studies measuring dehydration

Nineteen studies measuring dehydration in an acute stroke population were included, however results were discussed from 20 studies, and only 18 were included in the data tables. Most studies used laboratory values as objective indirect diagnostic criteria, with blood urea nitrogen to creatinine ratio (BUN/Cr) and serum osmolarity being the most common laboratory markers used; only one study used patient weight as a measure. Within the twelve studies that used BUN/Cr there were three different definitions of dehydration. Overall rates of dehydration in the acute stroke population ranged from 29 to 70%.

### Studies evaluating treatments of dehydration

Two comprehensive Cochrane reviews (Chang and Jensen, 2014; Visvanathan et al. 2015) exploring this research area were published shortly before this review was completed. The reviewers therefore included only five studies published after the Cochrane papers. Although four studies (Lin et al. 2015; Lin et al. 2015; Lin et al. 2016; Mucke et al. 2012) suggested that treatment of dehydration could improve function and lower death rate, the remaining study (Dharmasaroja, 2016) suggested that high volumes of rehydration in patients with large strokes may increase cerebral oedema.

### Outcomes after stroke in dehydrated patients

Outcome measures across the studies varied but included death, dependency, early neurological deterioration (END), stroke in evolution (SIE), hemispatial neglect, and discharge to nursing home. The review stated that all clinical studies of dehydration measures at the time of stroke reported worse clinical outcomes in dehydrated patients. However, not all studies included in the review measured patient outcomes. Nevertheless, where patient outcomes were assessed, the majority were found to be poorer in those classified as dehydrated (see Table 1).

### Biological mechanisms using animal models to investigate the relationship between dehydration and stroke

The review concluded, based on four animal studies which were not reported in the tables of included studies but discussed in the narrative results section, that poor hydration status is associated with worse outcomes. One animal study showed that supported access to food and drink was independently associated with decreased mortality regardless of infarct size (Lourbopoulos et al. 2017).

## Commentary

### Critical appraisal

Using the Joanna Briggs Institute Critical Appraisal tool for systematic reviews (Aromataris et al. 2015), we determined that 5 of the 11 criteria were deemed satisfactory (see Table 2). The review provides a satisfactory overview of the research to date, but this must be interpreted within the context of the six quality criteria that were not met or lacked clarity. While the critical appraisal criteria chosen by the review’s authors were appropriate, only one reviewer appraised the eligible studies. Best practice in conducting a systematic review requires two or more reviewers to undertake critical appraisal, neglecting this introduces the potential for error and reduces confidence in the review findings. No methods to minimise errors in data extraction were reported and the likelihood of publication bias was not discussed. Finally, while the recommendations for research were extensive, the recommendations for practice, such as utilising hydration therapy with isotonic fluids, were not supported by the reported data, limiting interpretability for healthcare practitioners.

Based on the critical appraisal, the review did not comprehensively attempt to minimise bias in the study selection, data extraction process or critical appraisal of included studies. Consequently, the validity and reliability of the synthesis may be limited in its implications for practice.

### Implications for practice

Overall, the findings of the review highlight that dehydration may be a substantial problem impacting 29% to 70% of stroke patients (Bahouth et al. 2018). This variation in the rates of dehydration reported in the included studies may be partially explained by the variety and range of measurement techniques utilised in the study designs. The heterogenous nature of the evidence base limits the opportunity for comparisons to be made across studies, and therefore the development of meaningful recommendations to improve practice.

Although the review aimed to standardise terminology and identify gaps in the literature, these were not covered within the results section. This omission in reporting may be due to the limited number of studies, and the heterogeneity of those that exist, resulting in a lack of data to achieve the review aims. Despite the inconsistencies in the review overall, the findings suggest an association between dehydration and poor outcomes in acute stroke.

In relation to clinical practice, the review highlights the detrimental effects of dehydration on patient outcomes (Bahouth et al. 2018. Although the interpretation of this evidence is limited by the review’s methodological limitations, the findings increase awareness of the impact of dehydration among this population for healthcare practitioners. This increased awareness may allow for early identification and prompt management of dehydration in these patients. That said, further research is needed to recommend a specific clinical assessment given that there is a dearth of evidence in this area (Oates and Price 2017). To minimise the acknowledged detrimental effects of dehydration, healthcare practitioners could incorporate routine screening for dehydration into their clinical assessments for patients presenting with acute stroke (Guastaferro et al. 2018; Miller et al. 2023).

The association between dehydration and poor outcomes in stroke may also prompt the development of educational initiatives and training programs for healthcare professionals (McCotter et al. 2016). Recent evidence suggests that continuing education programs, workshops, and conferences should emphasise the importance of hydration in acute stroke management (McCotter et al. 2016; Mullins 2021). By enhancing healthcare professionals' knowledge and skills in this area, they may be better equipped to identify and address dehydration more promptly and effectively (Miller 2023; Mullins 2021).

While the review highlights evidence suggesting rehydration therapies may improve clinical outcomes and functional independence (Lin et al. 2016), further robust research evidence is required to inform best practice in this area.

### Implications for future research

Bahouth et al. (2018) suggest that future research should focus on determining a single, validated, objective measure to clinically diagnose dehydration in stroke patients. They further recommend that the reporting of future research findings should include more detailed information about the type and severity of stroke, type and time point of dehydration measurement, and more clearly defined patient characteristics. An under researched area highlighted by the review was that of patient experience of dehydration after stroke, as no previous studies have explored this important aspect of care.

Further research could also investigate the underlying mechanisms linking dehydration and poor stroke outcomes, identify specific patient populations at higher risk, and evaluate the impact of hydration interventions on clinical outcomes. This research could contribute to an expanded evidence base, further informing clinical practice guidelines and fostering continuous improvement in stroke management.

### CPD reflective questions

1. What do you think the key take-away messages from the review are and why?
2. Are you satisfied with the way the authors conducted and reported the review? Justify your answer.
3. The authors concluded that a hydration therapy based on isotonic fluids could be promising. Do you agree based on the evidence presented? Justify your answer.

## Figures and Tables

**Table 1:** Characteristics of studies included in Bahouth et al. 2018 systematic review. (This table was amalgamated from analysis and/or narrative)

Author, year, country	Review Ref. No.	Review Table	Inclusion reason	Measure	% Dehydrated	Patient outcomes measured	Effect of dehydration	Observations
Akimoto et al, 2011, Japan	13	1	Measures Dehydration	BUN/Cr >25	29% (28/97)	No	N/A	Dehydration on admission is associated with higher prevalence of cardioembolic stroke
Bahouth et al, 2016, USA	35	1	Measures Dehydration	BUN/Cr >15 USG > 1.010	57% (114/201)	NIHSS Hemispatial neglect	Negative	Dehydration on admission is associated with more severe hemispatial neglect
Bhalla et al, 2000, UK	14	1	Measures Dehydration	pOsm >296mOsm/kg	NR	Death or dependency	Negative	Dehydration on admission is associated with increased mortality
Bhatia et al, 2015, India	15	1	Measures Dehydration	BUN/Cr >15 USG > 1.010	39% (45/114)	NIHSS END	Negative	Dehydration on admission is associated with early neurological deterioration
Chang et al, 2014, USA	42	Not in Table	Cochrane Review	-	-	-	-	Review showed no clear evidence of benefit of haemodilution therapy for ischaemic stroke
Chang et al, 2016, Taiwan	16	1	Measures Dehydration	BUN/Cr ≥15	70% (61/87)	NIHSS Collateral development	Negative	Dehydration on admission is associated with poor collateral flow development
Crary et al, 2013, USA	36	1	Measures Dehydration	BUN/Cr ≥15	53% (36/67)	No	N/A	Dehydration on admission with dysphagia is associated with worsened hydration status at discharge
Dharmasaroja, 2016, Thailand	30	2	Hydration Therapy	-	-	-	-	Higher volume of fluid intake is associated with increased brain oedema in cerebral infarction
Dehghani Firoozabadi, et al, 2013, Iran	17	1	Measures Dehydration	Increased BUN/Cr	NR	Death	Negative	Dehydration is associated with increased mortality
Furukawa et al, 2016, Japan	18	1	Measures Dehydration	Blood viscosity	NR	No	N/A	Dehydration is associated with the onset of ischaemic stroke (small artery occlusion SAO)
Gross et al, 2005, USA	39	Not in Table	Biological Model	-	-	-	-	Animal study: Many brain regions have depressed metabolism in chronic severe dehydration
Hyodo et al, 1989, USA	40	Not in Table	Biological Model	-	-	-	-	Animal study: Cerebral blood flow is increased by haemodilution in dogs with ischaemic stroke
Kafri et al, 2013, UK	29	Not in Table	Measures Dehydration	Bioelectrical Impedance	22% (6/27)	No	N/A	Bioelectrical Impedance Assessment appears ineffective at diagnosing water-loss dehydration after stroke
Lin CJ et al, 2016, Taiwan	31	2	Hydration Therapy	-	-	-	-	BUN/Cr based hydration therapy in ischemic stroke is associated with improved discharge outcomes
Lin LC et al, 2011, Taiwan	19	1	Measures Dehydration	BUN/Cr >15	15% (30/196)	NIHSS SIE	Negative	Dehydration on admission is associated with early clinical deterioration
Lin LC et al, 2011, Taiwan	20	1	Measures Dehydration	USG >1.010	56% (177/317)	NIHSS SIE	Negative	Dehydration on admission is associated with early clinical deterioration
Lin LC et al, 2014, Taiwan	32	2	Hydration Therapy	-	-	-	-	BUN/Cr based hydration therapy in ischemic stroke is associated with reduced occurrence of SIE
Lin WC et al, 2015, Taiwan	33	2	Hydration Therapy	-	-	-	-	BUN/Cr based hydration therapy in ischemic stroke is associated with decreased infections and LOS
Lip et al, 2002, UK	21	1	Measures Dehydration	Blood viscosity	NR	No	N/A	Explored haemorheology alterations in acute stroke. Abnormalities could not be linked to hydration status
Liu et al, 2014, Taiwan	22	1	Measures Dehydration	BUN/Cr ≥15	48% (1229/2570)	mRS BI	Negative	Dehydration on admission is associated with poor discharge outcomes
Lourbopoulos et al, 2017, Germany	37	Not in Table	Biological Model	-	-	-	-	Animal study: Ischaemic stroke mortality in mice is associated with inadequate food and/or water intake
Morris et al, 1999, USA	38	Not in Table	Biological Model	-	-	-	-	Animal study: Results demonstrate a differential response to dehydration in mice lacking AT1a receptors
Mucke et al, 2012, Germany	34	2	Hydration Therapy	-	-	-	-	Fluid intake > 2000 ml per day may prevent secondary stroke
Murray et al, 2015, Australia	23	1	Measures Dehydration	BUN/Cr >20	44% (35/79)	Adverse health outcomes	Unclear	Rehab patients, with and without dysphagia, with mobility issues may be at risk of dehydration
O’Neill et al, 1992, UK	24	1	Measures Dehydration	pOsm AVP	NR	Death or dependency	Negative	Increased AVP is associated with poor outcomes
Ott et al, 1974, Austria	46	Not in Table	Biological Model	-	-	-	-	Dehydration with atherosclerotic disease associated with high blood viscosity and may contribute to stroke
Rodriguez et al, 2009, USA	3	Not in Table	Measures Dehydration	Calculated pOsm		No	N/A	Dehydration is a potential contributing factor to the onset of ischaemic stroke
Rowat et al, 2011, UK	25	1	Measures Dehydration	U:C >60 Urine Colour >4	45% (9/20)	No	N/A	Further research is needed to develop a practical tool for the prevention, detection, and treatment of dehydration
Rowat et al, 2011, UK	26	1	Measures Dehydration	U:C >80	62% (1606/2591)	Death or dependency	Negative	Dehydration at any point during hospital stay is associated with poor discharge outcomes and death
Schrock et al, 2012, USA	27	1	Measures Dehydration	BUN/Cr >15	43% (138/324)	Death or dependency		Dehydration on admission is associated with poor discharge outcomes and death
Song et al, 2017, Korea	28	1	Measures Dehydration	Blood viscosity	NR	No	N/A	Dehydration is associated with the onset of ischaemic stroke (small artery occlusion SAO)
Visvanathan et al, 2015, UK	41	Not in Table	Cochrane Review	-	-	-	-	no evidence to guide the best volume, duration, or mode of parenteral fluid delivery for people with acute stroke

* **AVP** = Arginine vasopressin; **BI** = Barthel Index; **BUN/Cr** = Blood Urea Nitrogen to Serum Creatinine ratio; **END** = Early Neurological Deterioration; **LOS** = Length of stay; **mRS** = Modified Rankin Scale; **NIHSS** = NIH Stroke Scale; **NR** = Not reported; **pOsm** = plasma osmolality; **SIE** = Stroke in evolution; **Table 1**: Studies measuring dehydration in acute stroke (N=18); **Table 2**: Studies including recommended hydration therapies for acute stroke patients (N=5); **U:C** = Urea creatinine ratio; **USG** = Urine Specific Gravity

**Table 2:** Critical appraisal of Bahouth et al. 2018 using the JBI Checklist for Systematic Reviews and Research Syntheses.

JBI Critical Appraisal Checklist	Appraisal response
1. Is the review question clearly and explicitly stated?	No, the review question was not clearly stated.
2. Were the inclusion criteria appropriate for the review question?	Yes, the review stated a broad inclusion criteria.
3. Was the search strategy appropriate?	No, there was insufficient detail reported to assess the appropriateness of the strategy.
4. Were the sources and resources used to search for studies adequate?	Yes, a systematic literature search was conducted from three bibliographic databases
5. Were the criteria for appraising studies appropriate?	Yes, appraisal was conducted using a validated tool (QATQS).
6. Was critical appraisal conducted by two or more reviewers independently?	No, critical appraisal of included studies was undertaken by only one reviewer.
7. Were there methods to minimize errors in data extraction?	No, the process of data extraction was not clearly stated.
8. Were the methods used to combine studies appropriate?	Yes, it appears a narrative synthesis was conducted on heterogenous literature.
9. Was the likelihood of publication bias assessed?	No, the review did not explore publication bias.
10. Were recommendations for policy and/or practice supported by the reported data?	No, the recommendations for policy and/or practice were not clear.
11. Were the specific directives for new research appropriate?	Yes, the review makes clear recommendations for future research.

## Key Points

1. There is currently no gold standard of measurement of dehydration. Multiple definitions and diagnostic criteria have been used across a range of study designs.
2. Although there are limited studies investigating the effects of dehydration after acute stroke there appears to be an association with poorer outcomes.
3. Best practice remains unclear and further research exploring diagnostic criteria and rehydration therapies associated with stroke would improve the evidence base.
4. Dehydration is common at the time of stroke, often as a complication associated with swallowing difficulties (dysphagia), and management of hydration status is a crucial element of acute stroke care.
